# Impact on Public Health of the Spread of High-Level Resistance to Gentamicin and Vancomycin in Enterococci

**DOI:** 10.3389/fmicb.2018.03073

**Published:** 2018-12-18

**Authors:** Mónica Sparo, Gaston Delpech, Natalia García Allende

**Affiliations:** ^1^Clinical Department, Medicine School, National University of Central Buenos Aires, Tandil, Argentina; ^2^Hospital Alemán, Buenos Aires, Argentina

**Keywords:** enterococci, clinical, foodborne, high-level resistance, gentamicin, vancomycin, clonal, transfer

## Abstract

Antibiotic resistance has turned into a global public health issue. Enterococci are intrinsically resistant to many antimicrobials groups. These bacteria colonize dairy and meat products and integrate the autochthonous microbiota of mammal’s gastrointestinal tract. Over the last decades, detection of *vanA* genotype in *Enterococcus faecium* from animals and from food of animal origin has been reported. Vancomycin-resistant *E. faecium* has become a prevalent nosocomial pathogen. Hospitalized patients are frequently treated with broad-spectrum antimicrobials and this leads to an increase in the presence of VanA or VanB vancomycin-resistant enterococci in patients’ gastrointestinal tract and the risk of invasive infections. In humans, *E. faecium* is the main reservoir of VanA and VanB phenotypes. Acquisition of high-level aminoglycoside resistance is a significant therapeutic problem for patients with severe infections because it negates the synergistic effect between aminoglycosides and a cell-wall-active agent. The *aac(6′)-Ie-aph (2″)-Ia* gene is widely spread in *E. faecalis* and has been detected in strains of human origin and in the food of animal origin. Enzyme AAC(6*′*)-Ie-APH(2*″*)-Ia confers resistance to available aminoglycosides, except to streptomycin. Due to the fast dissemination of this genetic determinant, the impact of its horizontal transferability among enterococcal species from different origin has been considered. The extensive use of antibiotics in food-producing animals contributes to an increase in drug-resistant animal bacteria that can be transmitted to humans. Innovation is needed for the development of new antibacterial drugs and for the design of combination therapies with conventional antibiotics. Nowadays, semi-purified bacteriocins and probiotics are becoming an attractive alternative to the antibiotic in animal production. Therefore, a better understanding of a complex and relevant issue for Public Health such as high-level vancomycin and gentamicin resistance in enterococci and their impact is needed. Hence, it is necessary to consider the spread of *vanA E. faecium* and high-level gentamicin resistant *E. faecalis* strains of different origin in the environment, and also highlight the potential horizontal transferability of these resistance determinants to other bacteria.

## Introduction

Enterococci are resistant to diverse physicochemical conditions and are widespread in nature. They are capable of growing and surviving under harsh environmental conditions and have been found in soil, plants, birds, and insects (Butler, 2006; Ghosh and Zurek, 2015).

In the intestinal tract of humans and other animals, the genus *Enterococcus* can be found among their flora. The microbiological and ecological factors that contribute with intestinal colonization are unknown, even though up to 10^8^ CFU/g of enterococci have been found in human feces. In addition, strains from this genus have been isolated from fermented and dairy products. Moreover, some enterococcal strains have been regarded as food biopreservants and probiotics, although their safety remains questioned (Beibei et al., 2015).

Traditionally, enterococci have not been considered as community-acquired pathogens. Usually, these bacteria do not cause infectious diseases in healthy people, except for occasional urinary tract infections; however, *Enterococcus faecium* as well as *E. faecalis*, are prevalent producers of health-care associated opportunistic infections (Woodford and Livermore, 2009). The genomic plasticity of enterococci has contributed with their adaptation to the hospital environment. Their relevance as nosocomial-infections’ agents is bolstered by their natural resistance to multiple antimicrobials and an outstanding ability for acquiring and transferring genetic resistance determinants (Werner et al., 2013).

Enterococci express natural (intrinsic) resistance to antibiotics, e.g., clindamycin and trimethoprim-sulfamethoxazole. In addition, enterococci show a naturally low-level resistance to gentamicin. Minimum inhibitory concentration (MIC) values to gentamicin range from 6 to 48 μg/mL (Chow, 2000).

Antimicrobials consumption constitutes an important risk factor for colonization with multi-drug resistant enterococci because of the suppression of the competitive indigenous microbiota in the gastrointestinal tract. The increased number of gut enterococci, due to the decrease of competitive gut indigenous flora, frequently precedes bloodstream infections (Ubeda et al., 2010; Reyes et al., 2017).

Antimicrobials can be used in animal husbandry with therapeutic, prophylactic/metaphylactic and growth promotion purposes. Despite the use of antibiotics as growth promoters has been forbidden in many countries, worldwide, foods supplemented with antimicrobials are freely acquired in several countries with no veterinarian control, including in Argentina. This leads to bacterial exposure to sub-therapeutic concentrations of antibiotics and, hence, it may promote the expression of antibiotic resistance (Andersson and Hughes, 2014). Antimicrobials employed for human therapies and also used in animal production (in decreasing order) are tetracyclines, penicillins, macrolides, sulfonamides, aminoglycosides, lincosamides, and cephalosporins (Love et al., 2011; Kuehn, 2014). Specifically, ceftiofur, sulfamides and tetracyclines are used for prevention and treatment of pneumonia in pigs; gentamicin and neomycin are employed for the therapy of bacterial diarrhea (Dewey et al., 1999; EFSA, 2011).

The addition of antibiotics for growth promotion in animal feed became a common practice without rigorous testing. The mechanism of action in growth promotion induced by antibiotics appears to be related to the reduction of pathogenic bacteria in the intestines. The concentration of antimicrobials used for growth promotion has often been lower than that used for therapy and prophylaxis. These sub-therapeutic doses of antibiotics often create an auspicious condition for selecting antibiotic resistant bacteria (Van Immerseel et al., 2004; Dibner and Richards, 2005). Previously, McDonald et al. (2001) reported antimicrobial resistant enterococci in food produced with animals fed with antibiotics in sub-therapeutic doses.

Extensive use of antimicrobials in animal husbandry has exerted a considerable pressure for the genesis of antimicrobial-resistant bacteria in the environment, such as vancomycin-resistant enterococci (López et al., 2009; Ruzauskas et al., 2009; Marshall and Levy, 2011; Nieto-Arribas et al., 2011; Ribeiro et al., 2011; Sánchez Valenzuela et al., 2013).

Furthermore, enterococci, due to their characteristics of gastrointestinal colonization, environmental persistence, natural and acquired resistance to different antimicrobials and their availability to transfer genes horizontally, can be used as biomarkers of antimicrobial resistance in intensive husbandry.

## Transferable Genetic Determinants of Antimicrobial Resistance

Intensive breeding of animals, especially poultry, pigs and cattle, facilitates the selection, spread and resistance determinants transfer of resistant bacteria. Increased antimicrobials resistance in colonizing bacteria from animals and food of this origin was documented (Normanno et al., 2007).

The extended and permanent use of antimicrobials for therapy purposes and growth promotion purposes in husbandry contributed with drug-resistant bacteria selection in humans. When antimicrobials are used in low doses and in prolonged cycles, a selective pressure is exerted that favors the propagation of drug-resistant bacteria (Fey et al., 2000; Graveland et al., 2010).

As a result, antimicrobial-resistant enterococci, as well as other resistant gut bacteria, can be spread in the environment by fecal residues. These bacteria can rapidly transfer their resistance to other strains through genetic determinants carried by mobile elements. Resistant enterococci are able to persist in the animal intestine, contaminate the environment and food of animal origin, and transfer determinants to human gut’s isolates (Tasho and Cho, 2017). Moreover, community people can be exposed to antimicrobial resistant enterococci through direct contact.

Use of antimicrobials can enhance gene transfer between bacteria (Malhotra-Kumar et al., 2007). Gene conjugative transfer is frequent in the human gut, as well as in nature. Enterococci acquire antibiotic resistance genes, e.g., for high-level gentamicin resistance and glycopeptides resistance determinants (Willems et al., 2011; Sparo et al., 2012).

Further, enterococci can horizontally transfer resistance genes to relevant bacteria in clinical settings, such as *Escherichia coli*, *Staphylococcus aureus*, and *Listeria* spp. (Verraes et al., 2013).

Generally, severe infections caused by enterococci are treated with a cell-wall active agent-aminoglycoside (mostly gentamicin) combination. The emergence of β-lactam and glycopeptide resistance and high-level resistance to gentamicin in enterococci has led to the employment of alternative antimicrobials (Arias et al., 2010; Bartash and Nori, 2017).

**FIGURE 1:**
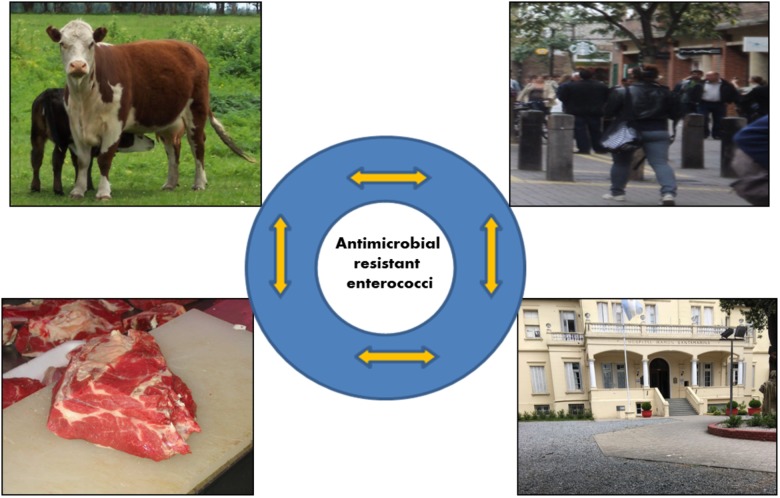
Bidirectional transfer of resistance determinants and/or resistant enterococci between different niches.

Figure 1 shows a presumable bidirectional transfer of resistance determinants and/or resistant enterococci between different niches such as human and animal. This transfer can occur through direct contact, foodborne contamination, as well as in health-care settings and the environment (community).

### High-Level Vancomycin Resistant Enterococci

In enterococci, vancomycin resistance is associated with different *van* genotypes each corresponding with a typical Van phenotype. These genes are chromosomal or extrachromosomal encoded in transposons and/or plasmids. In human *E. faecalis* and *E. faecium*, VanA and VanB (inducible resistance) are the most relevant types. *vanA* gene cluster is most often found on conjugative or non-conjugative plasmids (Cetinkaya et al., 2000; Top et al., 2008). VanA is encoded by Tn*1546*, or closely related transposons. *vanA* gene is linked with high-level resistance to vancomycin and teicoplanin, while variable-level resistance to vancomycin is associated with a VanB phenotype. The *vanB* operon is found among large conjugative plasmids or in the chromosome (Cetinkaya et al., 2000). The most frequent *vanB* subtype, *vanB2*, is encoded by conjugative transposons Tn*1549*-/Tn*5382*-like. It is interesting to note that Tn*1549-vanB* has also been detected in anaerobes that inhabit the human gut (Dahl et al., 2000; Launay et al., 2006).

VanA is the most prevalent glycopeptide resistance phenotype in *Enterococcus* linked with human infections, mainly expressed by *E. faecium* (Freitas et al., 2016). Lester et al. (2006) have proven, in volunteers, the existence of genetic transfer in the human intestine between ingested chicken *vanA*-*E. faecium* and non-resistant to vancomycin human *E. faecium*. It is important to highlight that this research has been performed in a human gut model with its complexity and its diverse microbiota.

Furthermore, there is a global concern regarding plasmid-mediated *vanA* transfer from *E. faecalis* to methicillin-resistant *S. aureus* and their co-colonization, with the likelihood of VanA-*S. aureus* isolation (Flannagan et al., 2003; Weigel et al., 2003).

In the last decades, *vanA*-*E. faecium* were recovered from animals and food of this origin. Initially, the European Union stated that there was a link between Veterinarian use of a glycopeptide (avoparcin) and the emergence of vancomycin resistance (Werner et al., 2008). After avoparcin’s ban, glycopeptide-resistance did not disappear. López et al. (2009) reported high-level vancomycin resistant enterococci (4%) from samples of animal origin 10 years after avoparcin was forbidden. Continuous presence of vancomycin-resistant enterococci in farms and in food of animal origin suggests that is possible the co-transfer of resistance genes located in the same conjugative plasmid, such as *vanA* and *ermB*, which encodes for macrolides resistance, widely used in Veterinary medicine. Also, the presence of ABC-type transporter genes and the toxin-antitoxin system may favor the persistence of vancomycin resistance determinants (Aarestrup, 2000). In addition, deficient hygiene conditions in animal husbandry, should not be underestimated (Garcia-Migura et al., 2007). In the same period, a different situation was observed in the United States, since food of animal origin glycopeptide-resistant *E. faecium* were not detected but, nevertheless, they emerged in health-care settings, turning into a pathogen almost as prevalent as *E. faecalis* had been so far (Coque et al., 1996; Ramsey and Zilberberg, 2009). However, in Michigan, United States, vanA-*E.faecium* was detected in farm animals where avoparcin was not used; which supports the existence of alternative ways for spreading of *van* genes, their transfer or carrying isolates from humans to animals (Johnsen et al., 2011; Gordoncillo et al., 2013).

In Argentina, *vanA*-*E. faecium* from artisanal food of animal origin was reported by Delpech et al. (2012). Previously, it was observed that animal-origin vancomycin-resistant *E. faecium* of animal origin were ingested in meats, proving the risk of resistant bacteria colonization when meat products carrying resistant bacteria were consumed (Heuer et al., 2006).

In Argentina, since the late 1990′s vancomycin-resistant *E. faecium* infections have been reported. In several Argentinean hospitals, the prevalence of clonal complex (CC) 17 carrying the *vanA* gene was detected. Most of these enterococci also expressed high-level aminoglycoside resistance (Corso et al., 2007).

Recently, during a year-period (2013), genetic relatedness (PFGE studies) between *vanA* enterococci from humans, food and the hospital environment in the District of Tandil (Argentina) was investigated. *vanA-E. faecium* (*n*: 13) were recovered from human, food and hospital environment samples. *van*A enterococci were distributed among seven clonal types; *esp* gene was detected in clinical strains. However, the clonal relationship between *vanA-E. faecium* of clinical and food origin was not found. The clonal relationship was observed among isolates from the hospital environment and from patients (Pourcel et al., 2017).

Bacterial conjugation provides an efficient gene transfer pathway and can be considered as the most relevant mechanism for the increase of antimicrobial resistance (Hammerum, 2012). It is possible that bacteria from food can constitute reservoirs of antimicrobial resistance.

The horizontal gene transfer of *vanA*-resistance between food strains and human gut microbiota becomes a possible mechanism of resistance dissemination when enterococci do not fit in the hospital settings (Hammerum et al., 2010).

### High-Level Gentamicin Resistant Enterococci

The most prevalent mechanism of high-level aminoglycoside resistance in clinical bacteria is their enzymatic modification. Three families of aminoglycoside modifying enzymes have been recognized: phosphotransferases (APH), acetyltransferases (AAC), and nucleotidyltransferases (ANT). Genes for aminoglycoside modifying enzymes are often plasmidic, with bacteria-bacteria aminoglycoside resistance dissemination (Bassenden et al., 2016).

The following risk factors for the acquisition of infections with high-level gentamicin resistant enterococci have been identified: previous long-term antimicrobial treatment, number of prescribed antimicrobials, previous surgeries, peri-operative antimicrobial prophylaxis, hospitalization term/antimicrobial treatment, urinary catheterization and renal failure. Infections caused by *E. faecalis* with HLGR constitute a severe risk for patients with invasive conditions and long-term hospitalization (Miranda et al., 2001; Wendelbo et al., 2003; Ceci et al., 2015).

The most ubiquitous HLGR gene among human and food enterococci is *aac (6′)-Ie-aph (2*″*)-Ia* that encodes AAC(6*′*)-APH(2″)-Ia, with acetyltransferase and phosphotransferase activities. Enterococci with this enzyme express resistance to most of the available aminoglycosides (MIC > 2,000 μg/mL), except for streptomycin (Leclercq et al., 1992). Generally, *aac(6′)-Ie-aph(2*″*)-Ia* gene is flanked by inverted repeats of IS256, composing transposon Tn*5281* in *E. faecalis* as part of a conjugative plasmid (Rosvoll et al., 2012).

Other monofunctional genes encoding aminoglycoside-modifying enzymes have been described, such as class APH (2″)-subclass I phosphotransferases, chromosomal [e.g., *aph(2*″*)-Ib* y *aph(2*″*)-Id*] and plasmidic [e.g., *aph(2*″*)-Ic*] genes. These resistance determinants were originally found on *Enterococcus* species different than *E. faecalis* and encode enzymes which confer resistance to gentamicin and amikacin. *aph(2*″*)-Ic* gene is associated with MIC for gentamicin ranging between 128 to 512 μg/mL. Nevertheless, *aph(2*″*)-Id* gene, initially described in human *E. casseliflavus*, is linked to HLGR. This gene has been detected in clinical vancomycin-resistant *E. faecalis* (Ramirez and Tolmasky, 2010; Economou et al., 2017).

From 2000 to 2002, in Denmark, the proportion of high-level gentamicin resistant *E. faecalis* isolates increased from 2 to 6% in the pig population. Simultaneously, an emergence of HLGR *E. faecalis* isolates among patients with infective endocarditis was detected in the North Denmark Region (DANMAP, 2002). Afterward, Larsen et al. (2010) demonstrated that all of these isolates (human and pig origin) belonged to the same clonal group, suggesting that pigs were a reservoir for high-level gentamicin resistant *E. faecalis* associated with enterococcal infections.

Sparo et al. (2012) proved the spread of enterococci with HLGR from animals to humans through the food chain, and also that enterococci isolated from food of animal origin and humans carried the same aminoglycosides resistant genes, as reported, also, by other authors (Hammerum et al., 2007).

Resistance to ampicillin and vancomycin is infrequent, although *E. faecalis* have been shown to acquire HLGR (Kuch et al., 2012). Recently, over a 1 year period, the presence of cytolysin and HLGR in *E. faecalis* from human (hospital), animal (chicken feces from a farm) and food (minced meat from shops) origin were studied. Clinical samples were obtained from patients with invasive infections in Hospital Ramón Santamarina from Tandil City, Buenos Aires Province (Argentina). In all enterococci with HLGR, *aac (6′)* *-Ie-aph (2″)-Ia* gene was amplified. *aac (6′)-Ie-aph (2″)-Ia* and *cylA* were detected in human, food and animal *E. faecalis*, proving its environmental spread (Sparo et al., 2013).

In patients presenting risk factors, a high-level intestinal colonization of *E. faecalis* can become a frequent precursor of human invasive infections by bacterial translocation. This event is favored by the enhanced employment of broad-spectrum antimicrobials that exert significant pressure over the intestinal microbiota, hence, resulting in a likely emergency of multi-resistant enterococci. The human gut is a considerable reservoir for microorganisms potentially capable of transfer resistance to conventional antimicrobials. Moreover, the fact that bacteria isolated from food of animal origin can behave as a resistance reservoir needs to be taken into consideration. *In vitro* studies performed to prove genetic exchange between enterococcal strains from humans and food of animal origin, are not conclusive (Sparo et al., 2012). Therefore, *in vivo* models for assessing genetic transfer are needed. Research carried out in animal models with their own microbiota it will not be able to reproduce the conditions of the human intestine. The use of human colon microbiota in germ-free mice is proposed as a model for reproducing the interaction between food strains and human gastrointestinal microbiota (Hirayama, 1999). Recently, HLGR determinants transfer from food to human bacteria was proven in an animal model. Immunocompetent BALB-C mice, colonized with human feces from an infant with no previous antimicrobial treatment, were used. This study showed evidence of the likelihood of high-level gentamicin resistance horizontal transfer from food to human *E. faecalis*. Therefore, a gene transfer model in non-sterile mice colonized with human gastrointestinal microbiota was standardized (Sparo et al., 2012).

It is needed to highlight that the rate of HLGR in vancomycin-resistant enterococci is higher than in vancomycin-susceptible enterococci strains. Mihajlović Ukropina et al. (2011) studied the frequency of antimicrobial resistance in enterococci isolated from blood cultures. HLGR was detected in vancomycin-resistant strains (87.6%) as well as in vancomycin-susceptible strains (9.9%). Hence, according to this study, HLGR in *E. faecium* is higher than in *E. faecalis*.

In an Argentinean study, *E. faecalis* strains with HLRG (*aac (6′)-Ie-aph (2″)-Ia* gene) and without glycopeptide resistance were recovered from human and food samples of animal origin. PFGE patterns showed four clonal types, and also that there was a clonal relationship between *E. faecalis* with HLGR isolated from food and those isolated from humans (Pourcel et al., 2017).

### Clonal Complexes of High-Level Vancomycin and Gentamicin Resistant Enterococci

Worldwide, MLST *E. faecium* data established that the majority of the clinical strains belong to the CC17, most of which are resistant to ciprofloxacin and ampicillin, and contain virulence genes. When new algorithms such as the Bayesian analysis of population structure (BAPS) were applied, it showed that CC17 consists of two large groups with different evolutionary origin: BAPS 2-1, containing sequence-type (ST) 78 and BAPS3-3 (ST17 and ST18). Most of the drug-resistant clinical isolates of hospital origin belong to both groups. The majority of community-origin isolates were grouped in the BAPS 2-1 group, genetically and evolutionarily different from hospital isolates and those of hospital origin are evolutionarily closer to those of farm animals. A similar trend was detected among vancomycin-resistant *E. faecium*, investigated in broiler flocks 15 years after the avoparcin ban, diversity was observed as well since they clustered in three BAPS populations (Willems et al., 2012; Bortolaia et al., 2015; Raven et al., 2016).

Several authors have highlighted the predominance of clonal lineages -17, -18 and -78 in human clinical isolates of *E. faecium*. It could be assumed that they have adapted to the intestinal environment and integrate their microbiota (Baquero and Coque, 2011; Faith et al., 2015; Tedim et al., 2015, 2017).

Nowadays, comparison of available genome sequences allowed to support the existence of two clades for *E. faecium*; one of the animal strains and hospital-associated enterococci (clade A) and another one of community strains (clade B), which includes human commensal isolates. Clade A has been subdivided into A1, including most of the clinical isolates (lineages ST17, ST18, and ST78) and A2, containing mainly strains of animal origin. It has also been shown that the genome of the strains included in the clade A1 has a larger size than those ones of strains belonging to A2, which seems to support the recent emergence of this clade and the importance of its recombination (Galloway-Peña et al., 2012; Tedim et al., 2015).

Unlike *E. faecium*, *E. faecalis* lack a clear structure in clades. Some clones are more frequent in hospitalized patients or in the community. Specifically, CC2 and CC9 both present high-level vancomycin resistance and have been described as highly risky due to their adaptation to the hospital environment and global dissemination (Freitas et al., 2009; Kuch et al., 2012; Guzman Prieto et al., 2016).

*E. faecalis* CC2, a high-risk CC, is frequently found among health-care associated isolates and represents hospital complexes linked with high-level aminoglycoside resistance (Weng et al., 2013). In addition, *E. faecalis* CC87, similar to CC2, expresses multi-drug resistance and can be associated with invasive infections (Ruiz-Garbajosa et al., 2006; Tedim et al., 2015).

## Impact in Human Infections and Therapeutic Options for Resistant Enterococci

Among bloodstream infection (BSI) associated with the healthcare environment, *Enterococci* is the third most common one. Although vancomycin-resistant enterococci have been clinically relevant pathogens for years, the majority of clinical data is retrospective (Wisplinghoff et al., 2004). Nowadays, vancomycin-resistant enterococci are the cause of one-third of all health care associated infections in the United States and one fifth in some European countries (Hidron et al., 2008; European Centre for Disease Prevention and Control [ECDC], 2010). Furthermore, mortality rates in patients with BSIs produced by vancomycin-resistant enterococci range between 20 and 46% (Han et al., 2009; McKinnell et al., 2011; Twilla et al., 2012).

Treatment of vancomycin-resistant enterococci’s BSI is particularly challenging. The therapeutic options include linezolid, daptomycin, quinupristin-dalfopristin, tigecycline, and lipoglycopeptides, such as telavancin, dalbavancin and oritavancin.

Due to limited clinical available data of lipoglycopeptides together with resistance issues in VanA enterococci, the role in systemic vancomycin-resistant enterococci infections for telavancin and dalbavancin is irrelevant. Oritavancin (the lipoglycopeptide with the broadest antibacterial coverage) has shown bactericidal activity against VanA and VanB vancomycin-resistant enterococci. This drug was approved for the treatment of acute bacterial skin infections and is currently undergoing clinical trials for the treatment of bacteremia (Zhanel et al., 2010; Messina et al., 2015).

In Europe, Teicoplanin can be used for VanB phenotype infections (Svetitsky et al., 2009).

Tigecycline has not been approved for the treatment of bacteremia because it does not achieve high serum concentrations. This tetracycline can be considered as one of the first-line treatments for polymicrobial intra-abdominal infections associated with vancomycin-resistant enterococci due to its high penetration into the peritoneal space (Arias et al., 2010).

Quinupristin-dalfopristin, effective only against *E. faecium*, has a high molecular weight, which renders it unable to cross the blood-brain barrier. This, added to the facts that it has frequent side effects and that it easily interacts with other drugs, limits its clinical use (Rubinstein et al., 1999).

Since approval, linezolid has been widely employed for vancomycin-resistant enterococci infections. The clinical success rate can vary based on the infection site and generally range between 50 and 80%. Lower success rates are generally seen in patients with bacteremia and infections without known focus (Birmingham et al., 2003; Kraft et al., 2012; Da Silva et al., 2014; Patel et al., 2016).

Linezolid has shown utility for treating infections by vancomycin-resistant enterococci non-susceptible to daptomycin. Surveillance analysis carried out in 2012 showed 99.5% susceptibility for linezolid against enterococci in the United States health systems (Mendes et al., 2014). Prolonged use of linezolid has been associated with resistance emergency (Pogue et al., 2007; McGregor et al., 2012).

Tedizolid is a next-generation parenteral and oral oxazolidinone with a broad spectrum bacteriostatic activity against resistant Gram-positive bacteria including VanA and VanB enterococci. It has been approved for the treatment of acute bacterial skin and soft tissues infections, and, currently, clinical trials for bacteremia and pneumonia treatment are being undergone (Rybak et al., 2014).

Daptomycin has been successful for multidrug-resistant enterococci and vancomycin-resistant enterococci infections’ treatment. Multiple analyses of the Cubicin Outcomes and Registry Experience (CORE) have shown a higher clinical success rate when used as first-line therapy for vancomycin-resistant enterococci bacteremia, 87–93% (Sakoulas et al., 2007; Mohr et al., 2009).

β-lactam antibiotics have been evaluated, *in vitro*, combined with daptomycin against vancomycin-resistant enterococci, including ampicillin, ceftaroline, ceftobiprole, and ceftriaxone, all of which produced synergistic effects even when β–lactam resistance was detected (Sakoulas et al., 2012, 2014; Hall Snyder et al., 2014; Werth et al., 2015).

For infectious endocarditis due to ampicillin susceptible and HLGR *E. faecalis*, ampicillin with ceftriaxone should be considered as an alternative treatment option, since it showed a similar efficacy to the observed ones for ampicillin with gentamicin, in susceptible strains, but with less nephrotoxicity. The saturation of several penicillin-binding proteins is the main reason why this combination presents a desirable bactericidal synergy (Mainardi et al., 1995; Murray, 2000; Fernández-Hidalgo et al., 2013; Economou et al., 2017).

### Alternatives/Complementary Therapeutic Options

Available evidence about infection control and prevention measures(ICP) to reduce vancomycin-resista t enterococci spread in adult hospitalized patients is insufficient. A systematic review published in 2014 (that included 9 studies with 30,949 participants) emphasized the importance of the implementation of hand hygiene program. A decrease of 47% in the vancomycin-resistant enterococci acquisition rate was observed when this measure is applied. Further studies with appropriate methodological design are urgently needed to define if ICP measures have an impact in reducing the acquisition of vancomycin-resistant enterococci among hospitalized patients (De Angelis et al., 2014).

A proposal for controlling antimicrobial resistance dissemination is to reduce antimicrobials employment in animal husbandry and promoting research of novel therapeutic alternatives. Probiotics are “living microorganisms which when administered in adequate amount confer a health benefit on the host” (Food and Agriculture Organization/World Health Organzation [FAO/WHO], 2001). These strains improve intestinal microbial balance, provide protection against gut pathogens and modulate the immune system. Probiotics are supplemented into animal feed (cattle, ducks, broilers, and chickens) and have beneficial effects on the food producing animals by enhancing weight gain, increasing egg/milk production, lowering the incidence of disease and mortality rates (Crittenden et al., 2005). Use of probiotics against pathogenic bacteria showed to be effective for reducing food-borne illnesses in consumers, in view of the absence of antibiotics in sub-therapeutic doses (Van Coillie et al., 2007).

A different approach is the use of microbial cell extracts that reduce the risks of bacterial translocation and infection (Sparo et al., 2014; Lemme-Dumit et al., 2018).

Bacteriocins are ribosomally synthesized peptides, with bacteriostatic/bactericidal activity, produced by various bacteria (Gálvez et al., 2007). The use of Gram-positive bacteriocins alone or in combination with antibiotics was proposed as a novel strategy to develop in human and veterinary medicines in order to help conventional antimicrobials against many multi-drug resistant pathogens. These combinations allow decreasing the MIC for achieving a bactericidal effect and, also, reduce undesirable side-effects of antibiotics (Lebel et al., 2013; Naghmouchi et al., 2013; Delpech et al., 2017). Randomized controlled trials are needed for obtaining scientific evidence about the usefulness of these novel compounds against pathogenic enterococci.

## Conclusion

Worldwide, enterococcal infections are among the most prevalent within those of nosocomial origin. Antimicrobial multi-resistant enterococci and their drug-resistant determinants spread by direct animal-human contact and/or through animal origin food. As mentioned above, the evidence is based on traditional microbiology and molecular tools, such as PFGE and MLST. Therefore, future studies combining phylogeographic methods with whole genomic sequence will provide reliable information for inferring bacteria movement between host populations.

Nowadays more countries are developing antibiotic-limiting policies, and thus arises a need of searching for an alternative or substitute for these drugs for sustainable food production, such as probiotics and bacteriocins.

## Author Contributions

MS and GD contributed to the writing of the microbiological and resistance aspects of the article, revised it and designed the Figure. NG contributed to the clinical and infectological aspects of the work.

## Conflict of Interest Statement

The authors declare that the research was conducted in the absence of any commercial or financial relationships that could be construed as a potential conflict of interest.
